# Direct and continuous dosing of propofol can saturate *Ex vivo* ECMO circuit to improve propofol recovery

**DOI:** 10.1051/ject/2023036

**Published:** 2023-12-15

**Authors:** Nitish Khurana, Till Sünner, Oliver Hubbard, Carina E. Imburgia, Venkata Yellepeddi, Hamidreza Ghandehari, Kevin M. Watt

**Affiliations:** 1 Utah Center for Nanomedicine, Department of Molecular Pharmaceutics, College of Pharmacy, University of Utah Salt Lake City Utah 84112 USA; 2 Philipps Universität Marburg, Institut für Pharmazeutische Technologie und Biopharmazie Robert-Koch-Straße 4 35037 Marburg Germany; 3 Department of Biomedical Engineering, College of Engineering, University of Utah 36 S. Wasatch Salt Lake City Utah 84112 USA; 4 Division of Clinical Pharmacology, Department of Pediatrics, School of Medicine, University of Utah 295 Chipeta Way Salt Lake City Utah 84108 USA

**Keywords:** ECMO, Propofol, Saturation, Dosing

## Abstract

*Background*: Extracorporeal membrane oxygenation (ECMO) is a cardiopulmonary bypass device that provides life-saving complete respiratory and cardiac support in patients with cardiorespiratory failure. The majority of drugs prescribed to patients on ECMO lack a dosing strategy optimized for ECMO patients. Several studies demonstrated that dosing is different in this population because the ECMO circuit components can adsorb drugs and affect drug exposure substantially. Saturation of ECMO circuit components by drug disposition has been posited but has not been proven. In this study, we have attempted to determine if propofol adsorption is saturable in *ex vivo* ECMO circuits. *Methods*: We injected *ex vivo* ECMO circuits with propofol, a drug that is highly adsorbed to the ECMO circuit components. Propofol was injected as a bolus dose (50 μg/mL) and a continuous infusion dose (6 mg/h) to investigate the saturation of the ECMO circuit. *Results*: After the bolus dose, only 27% of propofol was recovered after 30 minutes which is as expected. However, >80% propofol was recovered after the infusion dose which persisted even when the infusion dose was discontinued. *Conclusion*: Our results suggest that if ECMO circuits are dosed directly with propofol, drug adsorption can be eliminated as a cause for altered drug exposure.

**Field of Research**: Artificial Lung/ECMO

## Overview

Extracorporeal membrane oxygenator (ECMO) is a cardiopulmonary bypass device that provides complete respiratory and cardiac support in patients with severe cardiopulmonary failure and is a lifesaving technology for critically ill patients. Critically ill patients on ECMO are prescribed multiple drugs but the dosing for most drugs is unknown. Dosing for this population is different due to 1) direct adsorption of drugs by the circuit, 2) clearance by the circuits (e.g., hemofiltration), and 3) critical illness physiology which affects drug absorption, distribution, metabolism, and elimination [1, 2]. Altered drug pharmacokinetics (PK) can result in suboptimal dosing and place patients at risk for subtherapeutic exposure and toxicity [3].

Adsorption of the drug to circuit components is primarily driven by hydrophobic and electrostatic interactions [4]. Studies have suggested that this process might be saturable but this has never been proven [5, 6]. Propofol is a commonly used sedative that is highly lipophilic (log*P* = 3.8) and protein-bound (98%) and is known to be adsorbed by the ECMO circuit. Prior studies have demonstrated that up to 70% of propofol was adsorbed within 30 minutes of administering a bolus dose to the *ex vivo* ECMO circuit [7–9]. In this study, we repeated the single dose experiment and additionally gave a 2-hour infusion of propofol to determine if propofol adsorption was saturable in a pediatric *ex vivo* ECMO circuit.

## Description

ECMO circuits were assembled according to standard practice with a Quadrox iD oxygenator with bioline coating, Rotaflow R32 pump with bioline coating and custom perfusion tubing. Circuits were set up in a closed loop with an IV bag representing the “patient”. Circuits were primed with human-packed red blood cells (330 mL), fresh frozen plasma (150 mL), plasmalyte (100 mL), heparin (250 units), sodium bicarbonate (3.5 mEq), tromethamine (2 g), calcium gluconate (325 mg) and albumin (6.25 g) and adjusted to maintain physiological conditions. The flow was set to 1 L/min to mimic flows for a 10 kg child. For the control, 30 mL of primed blood was drawn from the ECMO circuit and transferred to a tube, and maintained at 37 °C in a water bath. Both the ECMO circuit and control were dosed with propofol at time 0 to achieve a target concentration of 50 μg/mL (for clinical relevance). Samples (1 mL) were collected at different time points (1, 5, 15, 30, 60, 120, 180, and 240 mins). After the 240-minute sample, the ECMO circuit was dosed with a continuous infusion of 6 mg/h representing the low end of dosing for a 10 kg child. The infusion was discontinued after 2 hours. Samples were collected at the following time points: 15, 30, 60, and 120 min after start of infusion and 1, 5, 15, 30, 60, 120, and 240 mins after discontinuing infusion. Collected blood samples were centrifuged immediately (@3000 rpm, 4 °C) and plasma samples were stored at −80 °C until ready for further analysis. The samples were then analyzed for propofol concentration using HPLC, and then converted to propofol remaining (propofol recovery) using the following equation:

(1) $$\mathrm{Recovery}\;(\%)\;=\frac{C_{t}}{C_{i}}\times 100,$$

where *C*_*t*_ is the concentration at time *t* and *C*_*i*_ is the concentration at time = 1 min for the bolus dose and *C*_*i*_ is the concentration at time = 15 mins for the infusion dose.

## Results

**Figure 1:**
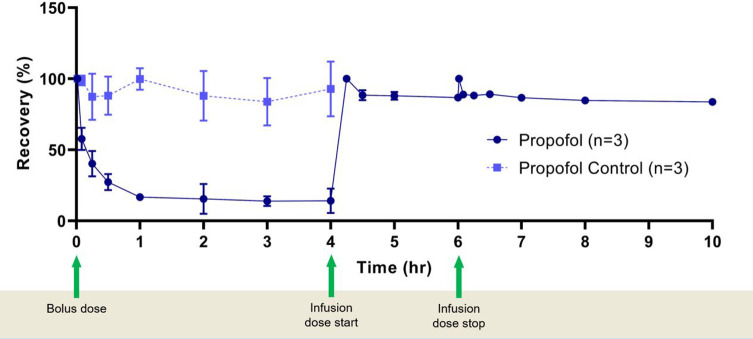
Recovery of propofol at different time points. Bolus dose was given at *t* = 0 and infusion dose started at *t* = 4 h and stopped at *t* = 6 h with samples collected up to a total of 10 h. Propofol represents samples collected from the ECMO circuit and propofol control represents the samples collected from non-ECMO falcon tube controls. Data represent Mean (*n* = 3) ± *SD.*

The percentage recovery of propofol was then plotted against time (Figure 1). All circuits and controls were run in triplicate.

For the bolus dose, the control demonstrated a very high recovery of propofol (above 80% at all time points) whereas in the ECMO circuit, a rapid decrease in propofol recovery was observed after the bolus dose, with a recovery of only 27% at 30 mins (Figure 1 and Table 1). However, when administered as a continuous infusion, propofol recovery was >80% during the infusion and that high level of recovery persisted even after the infusion was discontinued.

**Table 1:** Recovery of propofol at different time points.

Time point	Recovery (%) (Mean ± *SD*)	Control recovery (%) (Mean ± *SD*)	*P*-value
Bolus dose (*C*_*i*_ at 1 min)			
5 min	57.7 ± 7.8	98.9 ± 3.5	0.000558
15 min	40.3 ± 8.9	87.3 ± 16.2	0.005865
30 min	27.3 ± 5.6	88.1 ± 13.4	0.000962
1 h	16.7 ± 0.9	99.8 ± 7.6	0.000023
2 h	15.5 ± 10.5	88.0 ± 17.5	0.001791
3 h	13.9 ± 3.4	83.9 ± 16.8	0.001045
4 h	14.2 ± 8.5	92.8 ± 19.2	0.001483
Infusion dose started (*C*_*i*_ at 4 h 15 min)			
4 h 30 min	88.4 ± 3.6	NA	NA
5 h	88.1 ± 2.8	NA	NA
6 h	86.7 ± 2.7	NA	NA
Infusion dose ended (*C*_*i*_ at 6 h 1 min)			
6 h 5 min	89.0 ± 1.0	NA	NA
6 h 15 min	88.2 ± 1.1	NA	NA
6 h 30 min	89.1 ± 0.9	NA	NA
7 h	86.7 ± 1.1	NA	NA
8 h	84.7 ± 1.3	NA	NA
10 h	83.8 ± 0.6	NA	NA

## Discussion

Our results following a bolus dose of propofol are consistent with previous investigations on recovery of propofol [7–9]. As noted in those studies, approximately 70% of propofol is adsorbed on the ECMO circuit components after 30 mins of a bolus dose. Our results were consistent with previous studies as we observed 72% propofol to be absorbed on the ECMO circuit components after 30 mins of a bolus dose. Our results following a continuous infusion suggest that propofol adsorption is saturable when administered through the circuit. The saturation of the circuit can occur due to the hydrophobic interactions between the hydrophobic drug propofol and the hydrophobic material of the ECMO circuit components. The tubing of the ECMO circuit is made of poly(vinyl chloride) (PVC) and the oxygenators are made of poly(methyl pentene) (PMP), which are both hydrophobic. By saturating the circuit, propofol will then be available for its primary purpose: sedation of the patient. However, it is difficult to directly translate *ex vivo* results to a clinical setting because the experiments only quantify the drug-circuit interactions and it is unknown if propofol administered directly to the patient would achieve the same saturation. To address this, we have developed an approach that uses physiologically based pharmacokinetic modeling (PBPK) to translate *ex vivo* results into dosing recommendations. One limitation of this study is that we set up the circuit as we would for a pediatric patient and hence results may not translate directly to an adult circuit. However, although we used a pre-configured pediatric tubing set and set the flow rate to 1 L/min, our *ex vivo* circuit had an adult oxygenator (common practice in pediatric ECMO centers). Because adsorption is dependent in part on flow rate in an *ex vivo* circuit, in order to translate this to an adult ECMO circuit, higher doses of propofol would be required. Another potential limitation is that propofol rapidly distributes out of the plasma when administered to a patient, and it is unknown if those low concentrations in the plasma would be sufficient to saturate the circuit. There are some concerns that administering a lipophilic drug directly to an ECMO circuit can increase clotting and impair oxygenator function [10]. Another unknown is the possibility of the circuit acting as a reservoir and leading to the leaching of the drug back into circulation and causing toxicity [5]. Consequently, additional *ex vivo* and *in vivo* work is required before dosing propofol directly to the circuit can be routinely recommended.

## Conclusion

In summary, this is the first study to definitively show that adsorption by the ECMO circuit is saturable after an infusion dose is administered. Furthermore, these results demonstrate the potential of removing adsorption as a source of altered drug exposure by saturating the circuit. Additional work is needed to determine the optimal dose and if direct administration to the circuit impacts circuit function.

## Data Availability

The research data are available on request from the authors.
